# Functionalization and Self-Assembly of DNA Bidimensional Arrays

**DOI:** 10.3390/ijms12095641

**Published:** 2011-09-02

**Authors:** Alejandra V. Garibotti, Sónia Pérez-Rentero, Ramon Eritja

**Affiliations:** 1 Institute for Research in Biomedicine, Baldiri Reixac 10, E-08028 Barcelona, Spain; E-Mails: alejandra.garibotti@irbbarcelona.org (A.V.G.); sonia.perez@irbbarcelona.org (S.P.-R.); 2 Institute for Advanced Chemistry of Catalonia (IQAC), CSIC, CIBER-BBN Networking Centre on Bioengineering, Biomaterials and Nanomedicine, Jordi Girona 18-26, E-08034 Barcelona, Spain

**Keywords:** DNA tile, DNA bidimensional arrays, gold nanoparticles, self-assembly

## Abstract

Oligonucleotides carrying amino, thiol groups, as well as fluorescein, *c-myc* peptide sequence and nanogold at internal positions were prepared and used for the assembly of bidimensional DNA arrays.

## 1. Introduction

In recent years the use of the self-assembly properties of DNA to generate addressable nanostructures has been extensively investigated [1–3]. DNA has many physical and chemical properties that make it a useful molecule for the assembly of nanostructures [1–3]. The solid-phase synthesis and the PCR amplification methods have made long and complex DNA molecules readily available in abundant quantities [4]. The relatively high physicochemical stability makes DNA easier to handle in normal laboratory conditions [1–4]. Synthetic modification of the bases, sugars and 3′-5′-ends make it possible to add various functional groups at desired points [5].

A remarkable development in this field was the use of stable DNA Holliday junctions with addressable sticky ends to form two-dimensional DNA crystals [6]. The so-called “DNA-tile system” have since been used and adapted to generate systems with fine control of shape and function [6–10]. More recently, origami DNA has been developed allowing large 2D structures using single-stranded 7 kB long viral DNA held together by over 200 synthetic “staple” strands [11]. Origami DNA has been successfully assembled on photolitographically patterned substrates demonstrating a key step in the integration of bottom-up DNA assemblies with top-down microelectronics fabrication techniques [12].

We are interested in the site-specific functionalization of the DNA arrays with molecules and nanomaterials. In this direction, gold nanoparticles [13,14] and the *c*-*myc* [15] peptide have been introduced into the DNA tile system by using 5′-functionalized oligonucleotides. The resulting DNA-templated nanoparticle arrays are of potential interest in the manufacture of nanoscale integrated circuits for logic, memory, sensing and other applications [12,14]. Also the formation of DNA-templated peptide epitope arrays may find application in the development of nanosensors [15]. As the ends of the oligonucleotides are used in the assembly of the array, this strategy involves: (1) an oligonucleotide involved in the formation of the DNA arrays; and (2) the use of a complex annealing step to ensure the incorporation of the small and modified oligonucleotide to the DNA lattice.

Here we describe the formation of functionalized DNA arrays based on the Seeman’s DNA tile system [6] by direct functionalization of the internal positions of the oligonucleotides. Specifically, amino and thiol groups, as well as fluorescein, the *c-myc* peptide and nanogold were incorporated at selected internal positions of oligonucleotides. The resulting modified oligonucleotides were hybridized with the rest of the oligonucleotides yielding the desired DNA tiles. The amino and thiol groups have been selected as reactive groups in order to be further functionalized with compounds carrying carboxyl or maleimido groups. Fluorescein and the *c-myc* peptide sequence are model antigenic compounds that can be recognized by monoclonal antibodies. The DNA-templated array of fluorescein and the *c-myc* peptide antigens are intermediate steps for the fabrication of specific high-density monoclonal antibodies arrays which have potential interest for the development of nanobiochips [15]. Previously we demonstrated that chemical functionalization of DNA arrays with thiols direct the immobilization of DNA arrays to gold surfaces [16]. Here we demonstrate that DNA arrays can be used to template large ordered arrays of specific molecules of interest.

## 2. Results and Discussion

**Figure 1:**
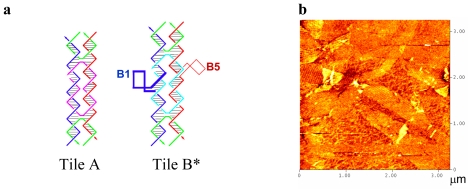

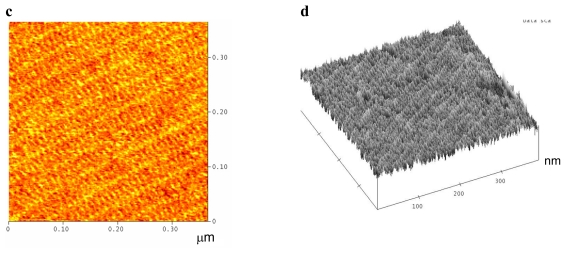
The A-B* DNA tile system [6]. (**a**) Each tile is formed by 5 oligonucleotides that are represented in different colors. (**b**) Topological AFM image of the A-B* DNA lattice assembled on mica. Brighter (taller) diagonal ridges correspond to rows of the topological marker (hairpins present on tile B*) and fainter bands to DNA tile A. (**c**) Detailed AFM image of a small area of the DNA array. (**d**) 3-D image of the AFM image shown in Figure 1(c) showing the lines of protruding hairpins used as topological markers. The apexes of these protruding hairpins have been selected to introduce selected chemical groups.

The aim of this work was the preparation of modified 2D DNA arrays. We focused on the A-B* tile system described by Winfree *et al.* [6] which comprises two DNA tiles, A and B*. Each tile is formed by the assembly of 5 oligonucleotides (A1–A5, B1–B5, Table 1). Tile B* has two loops protruding out of the plane of the DNA lattice in opposite directions. These two loops were used as topographic labels to indicate the position of tile B* on the DNA lattice during the process of visualization by atomic force microscopy (AFM). Figure 1 shows the formation of the unmodified DNA array using A-B* tile system [6]. As observed in Figure 1 (b) large arrays are formed which occupies a few micrometers. The position of the protruding DNA loops is clearly observed by AFM. We selected these loops for the introduction of the modifications as these loops are external and contain 4 unpaired thymidines, ideal for the introduction of the modifications.

### 2.1. Amino-Modified DNA Arrays

**Scheme 1:**
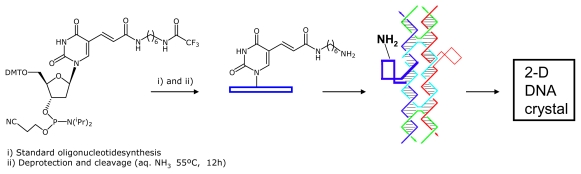
Steps used in the formation of the amino-modified A-B* DNA array. A phosphoramidite of the thymidine carrying an amino group is used to introduce the amino group at one specific site at the apex of one of the topological loops of tile B* described by Winfree *et al.* [6]. Annealing of the appropriate oligonucleotides yielded the desired DNA lattice that was then deposited on mica substrates.

Oligonucleotide **11** carrying one single amino group at the apex of the topological loops was prepared as shown in Scheme 1. Amino groups were selected due to their special reactivity as nucleophiles. This reactivity may be used for further functionalization with compounds carrying carboxyl groups. A phosphoramidite derivative of 2′-deoxyuracil carrying an amino alkyl group at position 5 of the uracil nucleobase was used to introduce the amino group at one specific site of oligonucleotide B1 (Table 1). The site of modification was one of thymidines forming the unpaired loop at the apex of one of the topological loops of tile B*. First a 20-base oligonucleotide containing 5 amino-dT residues was prepared and the stability of this analogue in oligonucleotide synthesis conditions was confirmed by mass spectrometry analysis (Supplementary data). Then, oligonucleotide sequence **11** was synthesized and purified by denaturing gel electrophoresis. The purified oligonucleotide was hybridized with the rest of the oligonucleotides forming tile B* (B2–B4). DNA tile B* carrying one single amino group at the unpaired loop position was able to form correct B* DNA tile as observed by native gel electrophoresis analysis.

**Figure 2:**
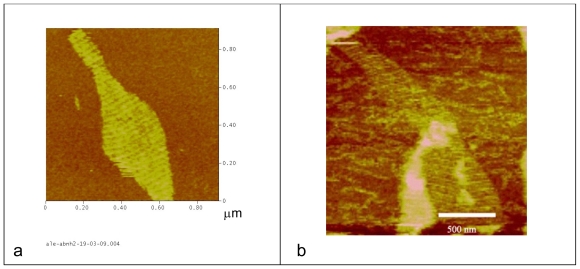
(**a**) Topological AFM image of the A-B* DNA lattice carrying amino groups assembled on mica. Brighter (taller) diagonal ridges correspond to rows of the topological marker (hairpins present on tile B*) and fainter bands to DNA tile A. (**b**) Topological AFM image of the A-B* DNA lattice carrying fluorescein groups assembled on mica.

Next, oligonucleotide sequence **11** was hybridized with the rest of the oligonucleotides A1-A5 (**1– 5**) and B2-B4 (**7–10**) yielding the desired DNA array that was then deposited on mica substrates. As can be seen from the AFM image in Figure 2a the DNA lattice carrying an amino group at the topological loop was observed on mica. The spacing of the decorated columns was 32 ± 1 nm, which was the same value observed in the bibliography for unmodified DNA lattices [6].

### 2.2. Fluorescein-, c-myc Peptide- and Nanogold-Modified DNA Arrays

**Scheme 2:**
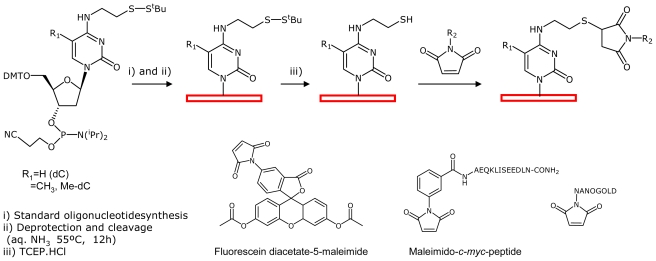
Synthesis of oligonucleotides carrying fluorescein, *c-myc* peptide and nanogold at the apex of one of the topological loops of tile B* described by Winfree *et al.* [6].

Recently, the chemical functionalization of DNA arrays with thiols has been demonstrated [16]. The introduction of the thiol groups in the B1 and B5 DNA oligonucleotides was done using the strategy described above for the introduction of amino groups (Scheme 1). We used cytosine phosphoramidite derivatives for the introduction of the thiol groups protected with the *t*-butylthio group (Scheme 2). The use of the protected form of the thiol groups avoids the formation of undesired disulfide bonds. We modified tile B* by introducing one *N*^4^-[(*t*-butyldithio)ethyl]-dC or one *N*^4^-[(*t*-butyldithio)ethyl]-5- methyl-dC residue to replace one thymidine residue at the unpaired loop positions of the oligodeoxynucleotides (Scheme 2). This involved preparing the two longer oligonucleotides of tile B* (69, 70 bases), which carry one single one *N*^4^-[(*t*-butyldithio)ethyl]-dC or one *t*-butyldithio-ethyl-5- methyl-dC residue in the middle of the sequence. The appropriate phosphoramidites were synthesized as described [17,18]. Oligonucleotide sequences carrying thiol groups protected with the *t*-butylthio group (**12**, **13**, Table 1) were purified by denaturing polyacrylamide gel electrophoresis (PAGE). The formation of the correct thiol-modified DNA arrays on mica have already been described [16].

Next, we prepared oligonucleotides carrying fluorescein, *c-myc* peptide and nanogold at the apex of the topological loops (Scheme 2). Fluorescein and the *c-myc* peptide sequence were selected because they are model antigenic compounds that can be recognized by monoclonal antibodies. Nanogold was selected as model gold nanoparticle. Maleimido derivatives of fluorescein and nanogold are commercially available. Maleimido- *c-myc* peptide was synthesized as described [17,18].

The synthesis of oligonucleotide conjugates carrying fluorescein (**14**) or *c-myc* peptide (**15**) started with the synthesis of thiolated oligonucleotide B5 (**13**, Table 1). This oligonucleotide was treated with tris(carboxyethyl)phosphine to remove the *t*-butylthio protecting group. The corresponding free thiol oligonucleotide was reacted with the corresponding maleimido derivatives as described [17–20] (Scheme 2) to yield the desired conjugates (**14**–**15**). Fluorescein and peptide conjugates **14** and **15** were purified by gel electrophoresis (supplementary information). As oligonucleotides **14** and **15** are too long for mass spectrometry analysis, we set up the conjugation reactions with a shorter DNA sequence and the corresponding fluorescein and *c*-*myc* conjugates were obtained in good yields. The desired conjugates were characterized by mass spectrometry (supplementary information).

The preparation of the nanogold conjugate **16** was slightly different. First thiolated oligonucleotide B1 (**12**, Table 1) was synthesized and purified by gel electrophoresis. The purified oligonucleotide **12** was treated with 0.1 M dithiothreitol (DTT) overnight at 50 °C to remove the *t*-butylthio protecting group and the resulting thiolated oligonucleotide was incubated with maleimido-Nanogold. The resulting nanogold conjugate **16** was used directly from the conjugation reaction without further purification. The functionalization of the Nanogold with the desired oligonucleotide was confirmed by agarose gel electrophoresis [21].

**Figure 3:**
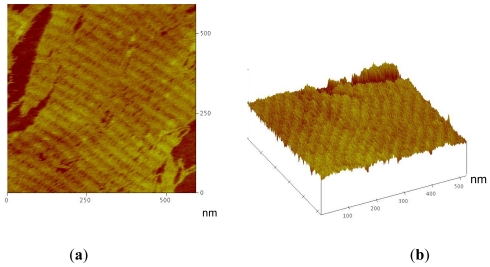
(**a**) Topological AFM image of the A-B* DNA lattice carrying nanogold assembled on mica. Brighter (taller) diagonal ridges correspond to rows of the topological marker (hairpins present on tile B*) and fainter bands to DNA tile A. (**b**) 3-D image of the AFM image shown in 5 (a) showing the position of the topological markers.

DNA tiles B* formed with these conjugates were able to form correct tiles as observed by gel electrophoresis analysis of hybridized equimolar mixtures of the corresponding B1-B5 oligonucleotides (supplementary information). Figure 2b shows the DNA lattices carrying fluorescein formed on mica. Figure 3 shows the arrays formed using nanogold-oligonucleotide conjugate **16**. Nanogold was not observed as the size (around 1 nm) is similar to the size of double-stranded DNA. The observation of DNA arrays using peptide-oligonucleotide conjugate **15** on mica did not yield positive results in spite of the good formation of B* tile observed by native gel electrophoresis. At present we do not know if the presence of the peptide sequence prevents the deposition of the array on mica or prevents the formation of large DNA arrays.

## 3. Experimental Section

### 3.1. Oligonucleotide Synthesis

Oligodeoxynucleotide sequences are shown in Table 1. The syntheses were performed on the Applied Biosystems Model 3400 DNA synthesizer using a scale of 0.2 μmol and standard 2-cyanoethyl phosphoramidites as monomers. The *N*^4^-[(*t*-butyldithio)ethyl]-2′-deoxycytidine and N^4^- [(*t*-butyldithio)ethyl]-5-methyl-2′-deoxycytidine phosphoramidites were prepared as described elsewhere [17,18]. After the addition of the *N*^4^-[(*t*-butyldithio)ethyl]-5-methyl-2′-deoxycytidine phosphoramidite, the oxidation solution used was a solution of *tert*-butyl hydroperoxide 10% in acetonitrile instead of the commercially available solution of iodine 0.02 M to avoid the oxidation of the thiol group described in reference [18]. The 5-amino-T-phosphoramidite was obtained from commercial sources. After the assembly of sequences, ammonia deprotection was performed overnight at 55 °C. Oligonucleotides were purified by polyacrylamide gel electrophoresis (PAGE) (see below). Purification of oligonucleotides by HPLC using DMT-on protocols yielded oligonucleotides that were not pure enough to generate complete DNA arrays (Supplementary section).

### 3.2. Preparation of DNA-Conjugates

Oligonucleotide sequence **13** was used to perform the reactions with fluorescein diacetate 5-maleimide, and maleimido *c-myc* peptide prepared as described [17]. For the preparation of each conjugate 50 OD_260_ units of crude oligonucleotide **13** were used. To cleave the disulfide bond, oligonucleotide **13** was dissolved in 1 mL of 0.1M triethylammonium acetate solution (pH = 7). Afterwards, 40 μL of a 0.5 M tris(2-carboxyethyl)phosphine hydrochloride (TCEP. HCl) solution were added to the solution and allow to react at 55 °C overnight. Under these conditions, the *tert*-butylthiol group was completely removed [18,19]. The resulting product was purified with Sephadex G-25 (NAP-10 column). The oligonucleotide carrying the free thiol group was eluted with 1.5 mL of sterile water.

Conjugation with fluorescein: To the resulting solution, 150 μL of a 1M triethylammonium acetate solution were added. Then, 100 μL of a solution 33 mM fluorescein diacetate 5-maleimide in DMF were added and allowed to react at room temperature overnight. The crude oligonucleotide was concentrated to dryness and then dissolved in 400 μL of a 0.2 M sodium hydrogen carbonate solution (pH = 9). The solution was heated at 55 °C for 1 h. Then 600 μL of sterile water were added and the excess of reagents and salts were removed by using a NAP-10 column. The oligonucleotide was eluted with 1.5 mL of sterile water and was purified by polyacrylamide gel electrophoresis (PAGE) (see below).

Conjugation with *c-myc*-peptide: To the resulting solution, 200 μL of a 1M triethylammonium acetate solution were added. Then, 500 μL of a solution 24 mM of the maleimido peptide [17,18,20] in sterile water were added. The pH was adjusted to 6 and the mixture was allowed to react at room temperature overnight. The crude oligonucleotide was concentrated to dryness and the excess of reagents and salts were removed by using a NAP-10 column. The oligonucleotide was eluted with 1.5 mL of sterile water and purified by polyacrylamide gel electrophoresis (PAGE) (see below).

Conjugation with maleimido-nanogold: Oligonucleotide sequence **12** was used to perform the conjugation with maleimido-Nanogold. First, thiolated oligonucleotide **12** was purified by polyacrylamide gel electrophoresis (PAGE) (see below). Then, the purified oligonucleotide was treated with 0.1 M dithiothreitol (DTT) overnight at 50 °C to remove the *t*-butylthio protecting group. The excess of DTT was removed by filtration over a NAP-10 column (Sephadex G-25). The desired thiol-oligonucleotide was eluted in 1 mL solution that was concentrated to 0.3 mL. The resulting thiolated oligonucleotide was incubated with commercially available maleimido-Nanogold. The resulting nanogold conjugate **16** was used directly from the conjugation reaction without further purification. The functionalization of the Nanogold with the desired oligonucleotide was confirmed by the presence of a brown spot in 3% agarose gel electrophoresis as described in reference [21].

### 3.3. Oligonucleotide Purification

Oligodeoxynucleotides were purified using denaturing gel electrophoresis. The gels contained 20% acrylamide (19:1, acrylamide/bisacrylamide) and 8.3 M urea, and were run at 55 °C on a Hoefer SE 600 electrophoresis unit. The running buffer comprised 89 mM Tris base, 89 mM boric acid, and 2 mM EDTA at pH 8.0. The sample buffer contained 10 mM NaOH, 1 mM EDTA, and a trace amount of Xylene Cyanol FF tracking dye. Gels were stained with ethidium bromide and the target band was excised and eluted in a solution containing 500 mM ammonium acetate, 10 mM magnesium acetate, and 1 mM EDTA. The eluates were extracted with *n*-butanol, which removes the ethidium bromide, followed by ethanol precipitation.

### 3.4. Formation of Hydrogen-Bonded Complexes and DNA Arrays

Complexes were formed by mixing a stoichiometric quantity of each strand, which was estimated by measuring the optical density at 260 nm. All 10 strands (A1-A5 and B1-B5) were mixed in 10 mM HEPES (pH 7.8), 12 mM MgCl_2_, and 2 mM EDTA. The final concentration of DNA was 0.2–0.4 μM. The final volume was 50 μL. Mixtures were annealed from 90 °C to room temperature for 40 h in a 2 litre water bath insulated in a styrofoam box. Previous to the array formation the correct assembly of tile A and modified tile B* was checked by nature PAGE.

### 3.5. Atomic Force Microscopy (AFM) Imaging

A sample of between 2–7 μL was spotted on freshly cleaved mica (Ted Pella, Inc.). The arrays were imaged in tapping mode in a buffer. The sample was deposited for 1–3 min and an additional 35 μL of fresh buffer was added to the liquid cell. All AFM imaging was performed on a NanoScope III (Digital Instruments) or Asylum MFP3D using commercial cantilevers with Si_3_N_4_ tips.

## 4. Conclusions

The preparation of long DNA sequences carrying thiol- and amino reactive groups in the internal positions is described. These oligonucleotides have been used for the preparation of oligonucleotide conjugates carrying fluorescein, peptides and nanogold at internal positions. The modified oligonucleotides were key elements for the formation of bidimensional DNA arrays carrying the molecule or nanomaterial of interest at the external B* loops. Although the observation of all the functionalized DNA arrays on mica have not been possible, we show that the long DNA sequences functionalized at a specific site can be prepared and the approach described is potentially interesting for the construction of DNA-mediated periodic arrays of selected molecules and nanomaterials at the nanoscale. The results described in this work may also be of interest for the preparation of functionalized DNA origamis [11,12]. This method relies on the use of a long single-stranded viral DNA and the length of the staple oligonucleotides is relatively short (between 30–40 bases) as well as the degree of purity is less demanding than the synthetic oligonucleotides used for the preparation of two-dimensional arrays. Work in this direction is currently ongoing.

## Supplementary Information



## Figures and Tables

**Table 1:** Sequences of oligonucleotides prepared.

#	Name	Sequence (5′-3′)
**1**	A1	GATGGCGACATCCTGCCGCTATGATTACACAGCCTGAGCATTGACAC
**2**	A2	GTAGCGCCGTTAGTGGATGTC
**3**	A3	TGTAGTATCGTGGCTGTGTAATCATAGCGGCACCAACTGGCA
**4**	A4	GACTGCGTGTCAATGCTCACCGATGCAACCAG
**5**	A5	CTGACGCTGGTTGCATCGGACGATACTACATGCCAGTTGGACTAACGG
**6**	B1	CGCTACCGTGCATCATGGACTAACCAGTGCTCGCTGATTTTTCAGCGAGTTACCGCATCGGACTCGGACAGCAGC
**7**	B2	CGTCAGGCTGCTGTGCTCGTGC
**8**	B3	AGTACAACGCCACCGATGCGGTCACTGGTTAGTGGATTGCGT
**9**	B4	GCCATCCGTCGATACGGCACCATGATGCACG
**10**	B5	GCAGTCGCACGACCTGGCGTCTGTTGGCTTTTGCCAACAGTTTGTACTACGCAATCCTGCCGTATCGACG
**11**	B1-amino	CGCTACCGTGCATCATGGACTAACCAGTGCTCGCTGATT**X**TTCAGCGAGTTACCGCATCGGACAGCAGC; **X** = amino-dT
**12**	B1-thiol	CGCTACCGTGCATCATGGACTAACCAGTGCTCGCTGATT**Y**TTCAGCGAGTTACCGCATCGGACAGCAGC, **Y** = *t*-butyldithio-ethyl-dC, *t*-butyldithio-ethyl-5-methyl-dC
**13**	B5-thiol	GCAGTCGCACGACCTGGCGTCTGTTGGCTT**Y**TGCCAACAGTTTGTACTACGCAATCCTGCCGTATCGACG, **Y** = *t*-butyldithio-ethyl-dC, *t*-butyldithio-ethyl-5-methyl-dC
**14**	B5-fluorescein	GCAGTCGCACGACCTGGCGTCTGTTGGCTT**Z**TGCCAACAGTTTGTACTACGCAATCCTGCCGTATCGACG; **Z** = *N-*(fluorescein-maleimido-*S*-ethyl)-5-methyl-dC
**15**	B5-*c-myc* peptide	GCAGTCGCACGACCTGGCGTCTGTTGGCTT**Z**TGCCAACAGTTTGTACTACGCAATCCTGCCGTATCGACG; **Z** = *N*-(*c-myc*-peptide-maleimido-S-ethyl)-5-methyl-dC; *c-myc* peptide sequence: Maleimido-Ala-Glu-Gln-Lys-Leu-Ile-Ser-Glu-Glu-Asp-Leu-Asn-CONH_2_
**16**	B1-Nanogold	CGCTACCGTGCATCATGGACTAACCAGTGCTCGCTGATT**Z**TTCAGCGAGTTACCGCATCGGACAGCAGC, **Z** = *N*-(Nanogold-maleimido-S-ethyl)-dC
