# Supplementary material for: Functionalization and Self-Assembly of DNA Bidimensional Arrays
Source: Int J Mol Sci. 2011 Sep 2;12(9):5641–51. doi: 10.3390/ijms12095641 (PMC3189739; doi:10.3390/ijms12095641)
Supplement: Supplementary file 1 [file ijms-12-05641-s001.pdf]

Supplementary Information

Alejandra V. Garibotti [1,2], Sónia Pérez-Rentero [1,2] and Ramon Eritja [1,2,*]

1 Institute for Research in Biomedicine, Baldiri Reixac 10, E-08028 Barcelona, Spain;
E-Mails: alejandra.garibotti@irbbarcelona.org (A.V.G.); sonia.perez@irbbarcelona.org (S.P.-R.)

2 Institute for Advanced Chemistry of Catalonia (IQAC), CSIC, CIBER-BBN Networking Centre on Bioengineering, Biomaterials and Nanomedicine, Jordi Girona 18-26, E-08034 Barcelona, Spain

* Author to whom correspondence should be addressed; E-Mail: recgma@cid.csic.es;
Tel.: +34-93-403-9942; Fax: +34-93-204-5904.

Received: 23 June 2011; in revised form: 12 August 2011 / Accepted: 23 August 2011 / Published: 2 September 2011

Abstract: Oligonucleotides carrying amino, thiol groups, as well as fluorescein, *c-myc* peptide sequence and nanogold at internal positions were prepared and used for the assembly of bidimensional DNA arrays.

Keywords: DNA tile; DNA bidimensional arrays; gold nanoparticles; self-assembly

---

Figure S1. 10% Denaturing (8M urea) polyacrylamide gel analysis of long oligonucleotides. Buffer conditions: 1 x Tris-borate-EDTA (TBE) buffer. The gel was run at 50 °C. Lane 1: 10-base pairs DNA marker. Lane 2: unmodified oligonucleotide B5, **10**. Lane 3: oligonucleotide B5 carrying *c-myc* peptide, **15**. Lane 4: unmodified oligonucleotide B1, **6**. Lane 5: oligonucleotide B5 carrying fluoresceine, **14**.

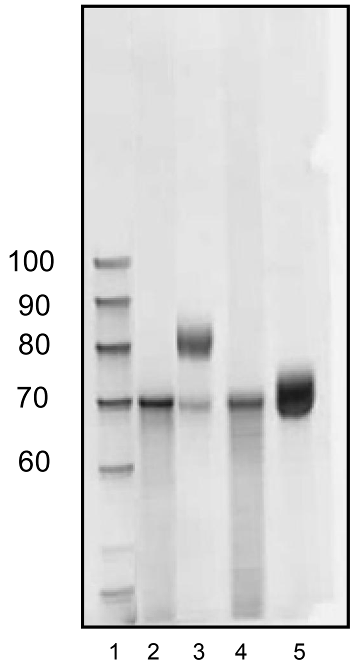

Figure S2. 8% Native polyacrylamide gel analysis of DNA-tile formation. Buffer conditions: TAE-Mg^{2+} buffer. The gel was run at 20 °C. Lane 1: 25-base pairs DNA marker. Lane 2: DNA-tile A (equimolar mixture of oligonucleotides A1-A5, **1-5**). Lane 3: DNA-tile B* (equimolar mixture of oligonucleotides B1-B5, **6-10**). Lane 4: DNA-tile B*-Fluoresceine (equimolar mixture of oligonucleotides B1-B4 and B5-fluoresceine, **6-9** and **14**). Lane 5: DNA-tile B*-*c-myc* peptide (equimolar mixture of oligonucleotides B1-B4 and B5- *c-myc* peptide, **6-9** and **15**).

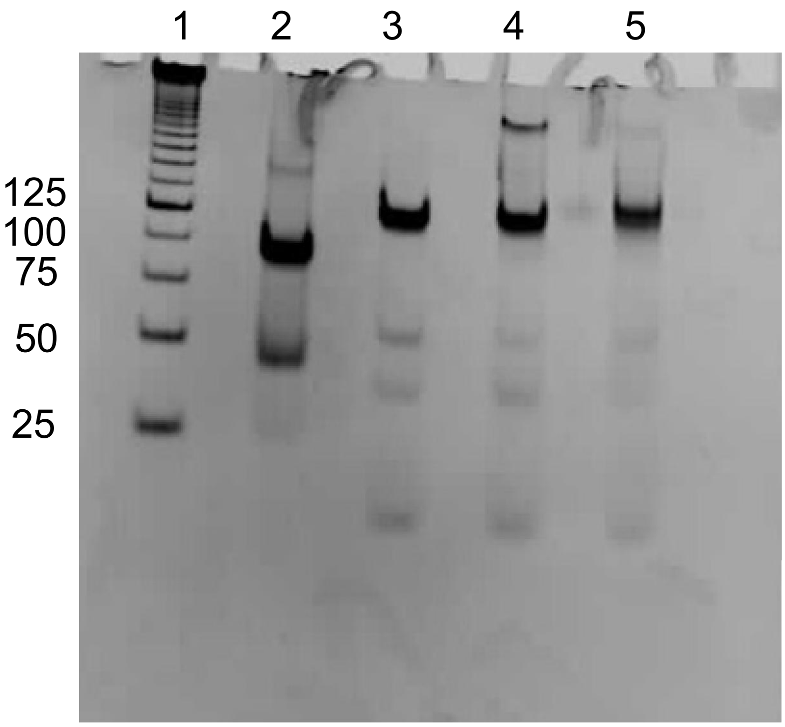

Figure S3. 3% Agarose gel of oligonucleotide-Nanogold conjugate, **16**. Buffer conditions: 0.5 x Tris-borate-EDTA (TBE) buffer. Lane 1: reaction of thiol-oligonucleotide **12** (B1-thiol) with maleimido-NANOGOLD after removal of the *t*-butylthio group. Lane 2: bromophenol blue and xylenecyanol dyes.

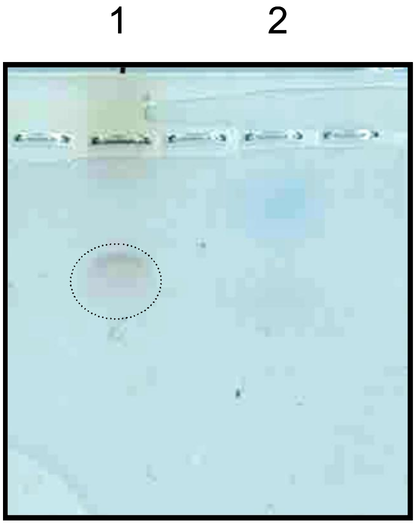

Figure S4. Influence of the purity of the oligonucleotides in the formation of the bidimensional DNA lattices.

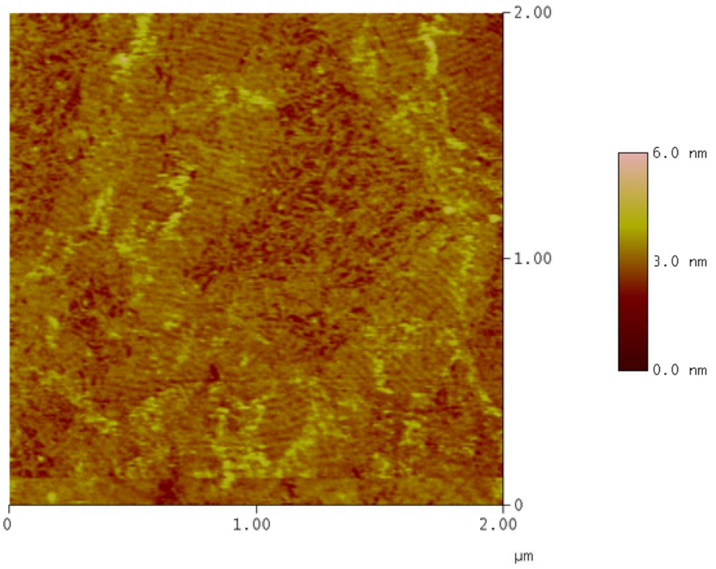

A) HPLC purified oligonucleotides (90–95% purity)

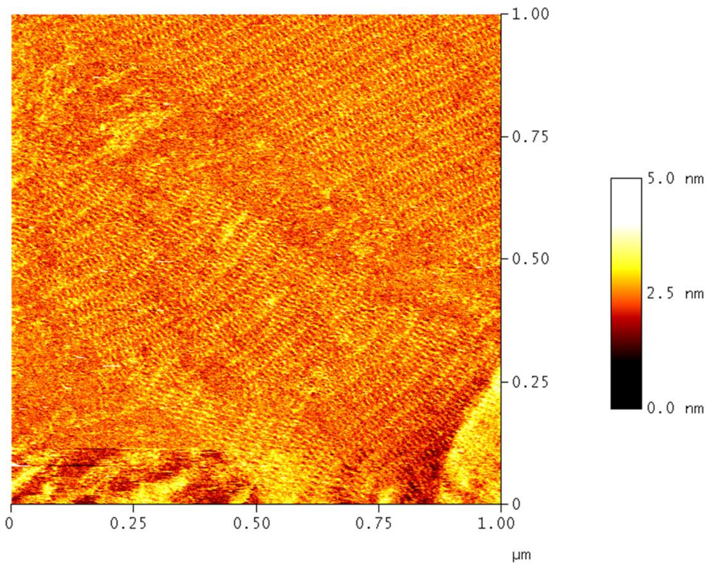

B) Gel-electrophoresis purified oligonucleotides (>99% purity)

Synthesis, Purification and Characterization a 20mer Oligonucleotide Carring Amino-dT Residues

The assembly of the sequence (5'-CGAG**X**CA**XX**GAG**X**CA**X**CGAG-3'; **X** = amino-dT) was performed using standard 200 nmol scale synthesis cycle (LV200). Ammonia deprotection was performed at 50 °C overnight. The resulting crude was analyzed by HPLC (Column: X-Bridge ^{TM}OST C_{18} (2.5 µm, 4.6 × 50 mm). Conditions: solvent A: 5% ACN in 100 mM triethylammonium acetate (pH = 7) and solvent B: 70% ACN in 100 mM triethylammonium acetate (pH = 7). Flow rate: 1 mL/min. 10 min linear gradient from 0–30% B. The main peak observed in the HPLC profile was also analyzed by mass spectrometry (MALDI-TOF). t_R = 5.7 min, MALDI-TOF m/z (negative mode $[M+H]^-$) calc for $C_{236}H_{316}N_{87}O_{123}P_{19}$ 6928.10, found 6927.50.

Figure S5. HPLC profile of a 20mer oligonucleotide carrying amino-dT residues.

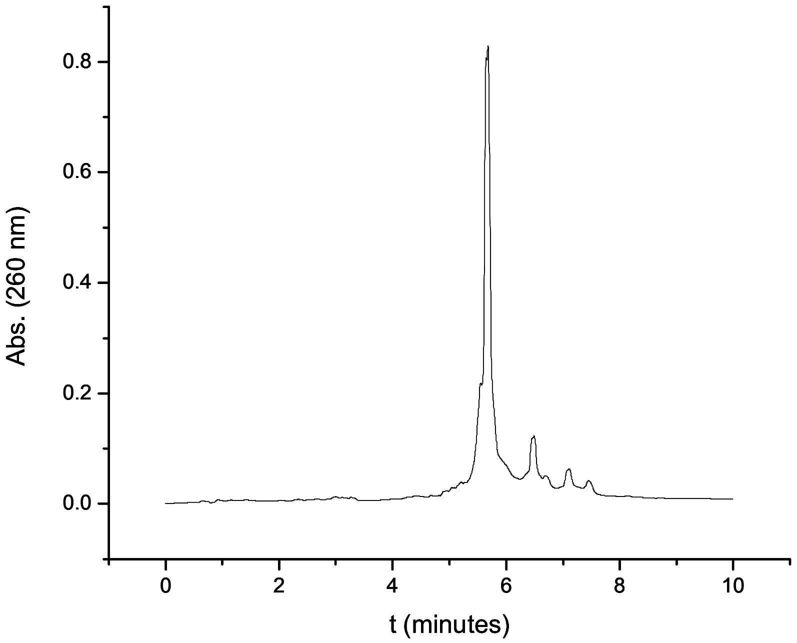

Figure S6. Mass spectrometry of a 20mer oligonucleotide carrying amino-dT residues.

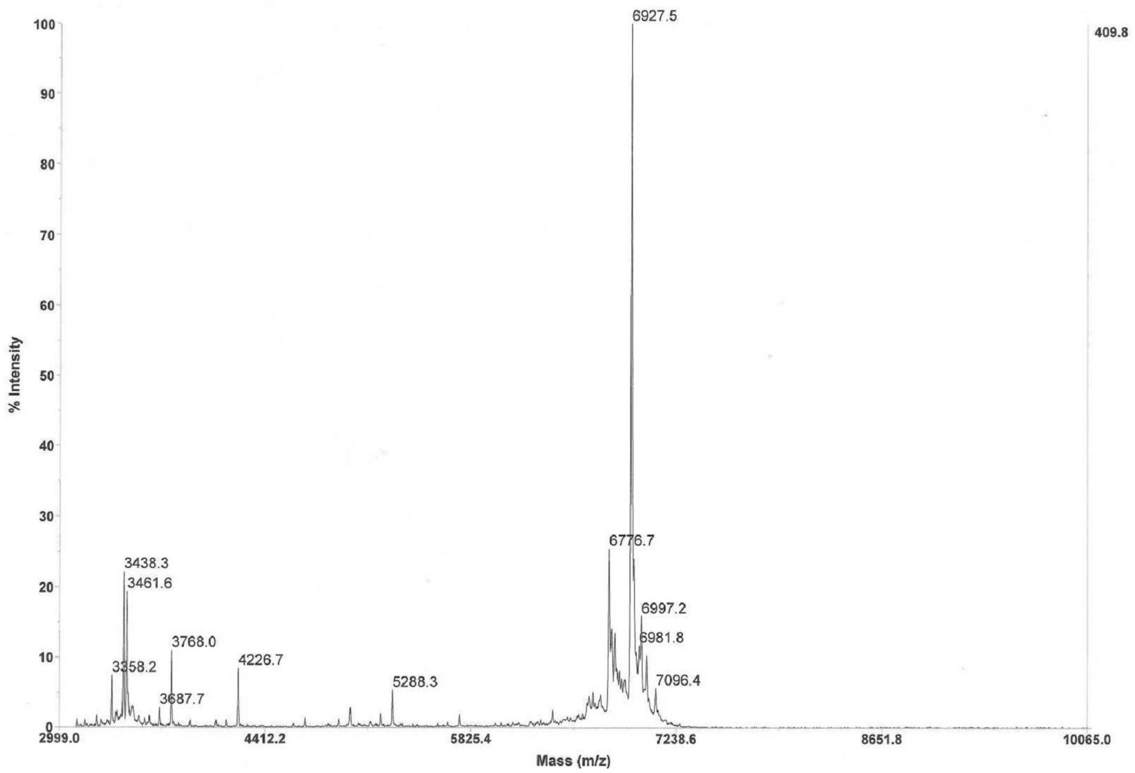

Mcalc.: 6928.10; Mfound: 6927.5

Synthesis, Purification and Characterization of a 13mer Carrying Fluoresceine and the c-myc Peptide

As oligonucleotides **14** and **15** are too long for mass spectrometry analysis we have prepared a 13mer DNA sequence carrying the *t*-butyldithio-ethyl-5-methyl-dC residue in an internal position of

the oligonucleotide as model compound to set up the conjugation reactions. The assembly of the sequence **17** (5'-TTCCA**Y**ATTACCG; **Y** = *t*-butyldithio-ethyl-5-methyl-dC) was performed as described in the experimental section. The resulting crude was analyzed by HPLC (Column: X-Bridge ^{TM}OST C_{18} (2.5 µm, 4.6 × 50 mm). Conditions: solvent A: 5% ACN in 100 mM triethylammonium acetate (pH = 7) and solvent B: 70% ACN in 100 mM triethylammonium acetate (pH = 7). Flow rate: 1 mL/min. 10 min linear gradient from 5–40% B. The main peak observed in the HPLC profile was analyzed by mass spectrometry (MALDI-TOF). t_R = 4.9 min, MALDI-TOF m/z (negative mode $[M+H]^-$) calc 4031.90, found 4032.52.

To cleave the disulfide bond, compound **17** was treated with tris(2-carboxyethyl)phosphine hydrochloride (TCEP.HCl) solution and allow to react at 55 °C overnight. Under these conditions, the *tert*-butylthiol group was completely removed yielding a new peak at 3.0 min (Figure S7B*)*.

Unprotected thiol oligonucleotide **17** was treated fluorescein diacetate 5-maleimide and maleimido-*N*-Ala-Glu-Gln-Lys-Leu-Ile-Ser-Glu-Glu-Asp-Leu-Asn-$CONH_2$ (*c-myc* peptide) as described in the experimental section yielding the desired conjugates **18** and **19** that were characterized by mass spectrometry (Figures S9-S12). Compound **18**: t_R = 3.6 min, MALDI-TOF m/z (negative mode $[M+H]^-$) calc for $C_{152}H_{180}N_{44}O_{84}P_{12}S$ 4372.08, found 4369.44. Compound **19**: t_R = 7.8 min, ESI-MS m/z (negative mode $[M+H]^-$) calc for $C_{197}H_{270}N_{61}O_{102}P_{12}S$ 5529.90, found 5531.93. Also found 5203.34, 4915.00, 4625.39, 4313.32, 4009.95, 3706.07, 3393.19 corresponding to different fragments of the oligonucleotide-peptide conjugate. Each fragment corresponded to the loss of one nucleobase, beginning at the 3'-end and the last fragment corresponded to the oligonucleotide-peptide conjugate having the modified nucleobase the 3'-end.

Table S1. Sequences of oligonucleotides prepared.

#	Sequence (5'-3')
**17**	TTCCA**Y**ATTACCG; **Y** = *t*-butyldithio-ethyl-5-methyl-dC
**18**	TTCCA**Z**ATTACCG; **Z** = *N*-(fluoresceine-maleimido-S-ethyl)-5-methyl-dC
**19**	TTCCA**Z**ATTACCG; **Z** = N(*c-myc*-peptide-maleimido-S-ethyl)-5-methyl-dC; *c-myc* peptide sequence: maleimido-Ala-Glu-Gln-Lys-Leu-Ile-Ser-Glu-Glu-Asp-Leu-Asn-$CONH_2$

Figure S7. HPLC profiles of **A)** protected oligonucleotide **17** and **B)** deprotected oligonucleotide **17**.

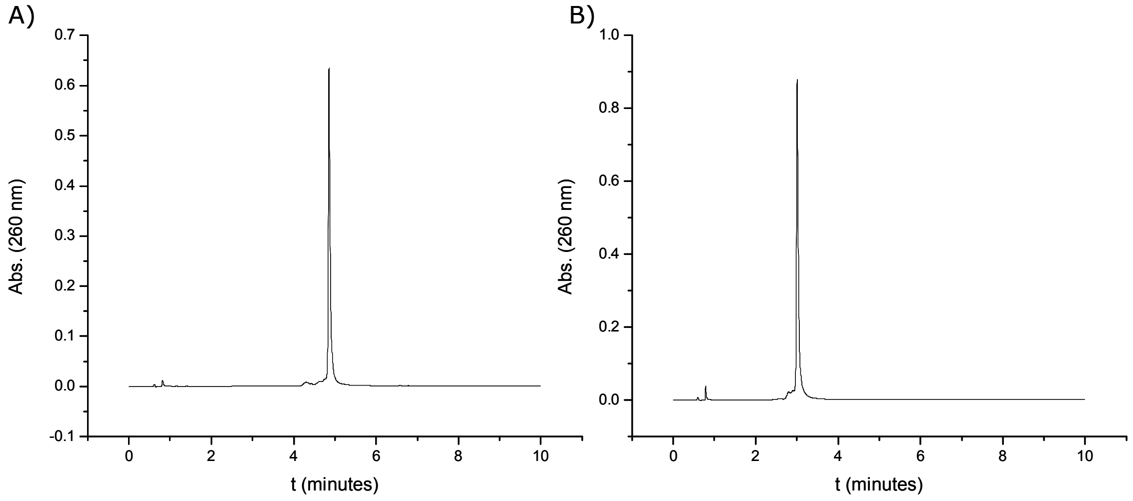

Figure S8. Mass spectrometry of compound **17**.

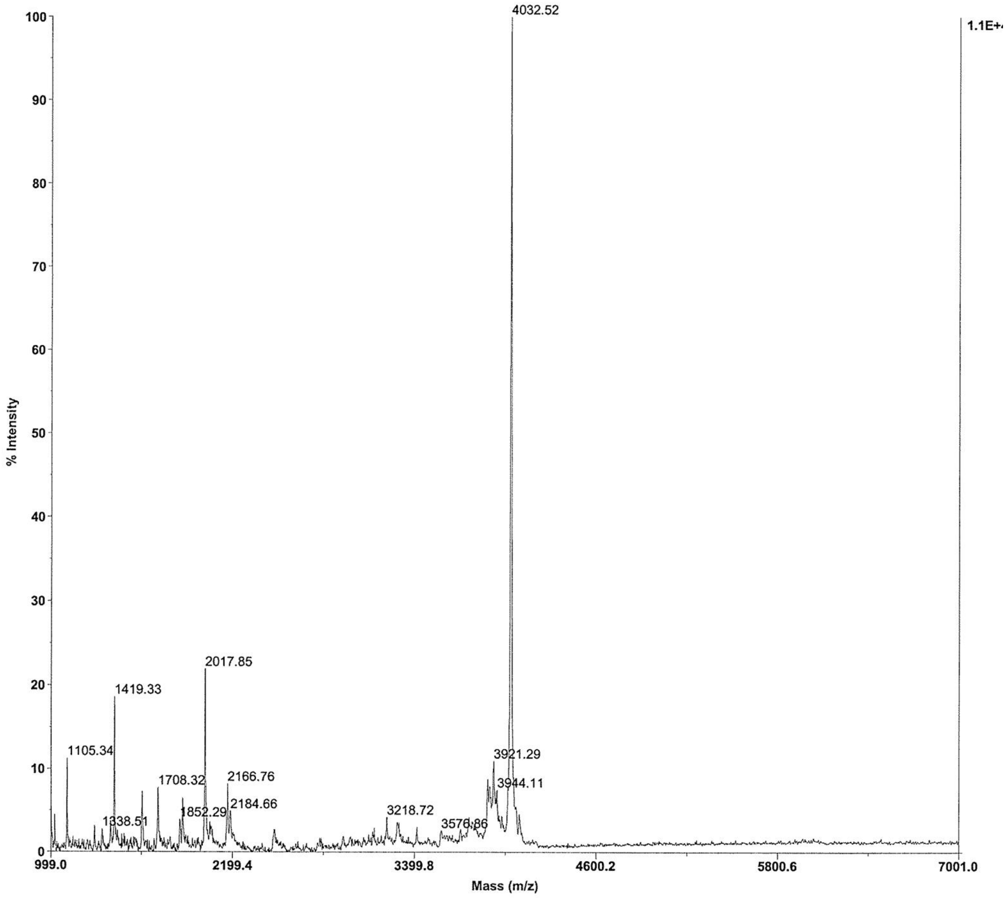

Mcalc.: 4031.90; Mfound: 4032.52

Figure S9. HPLC profile of fluoresceine conjugate **18**.

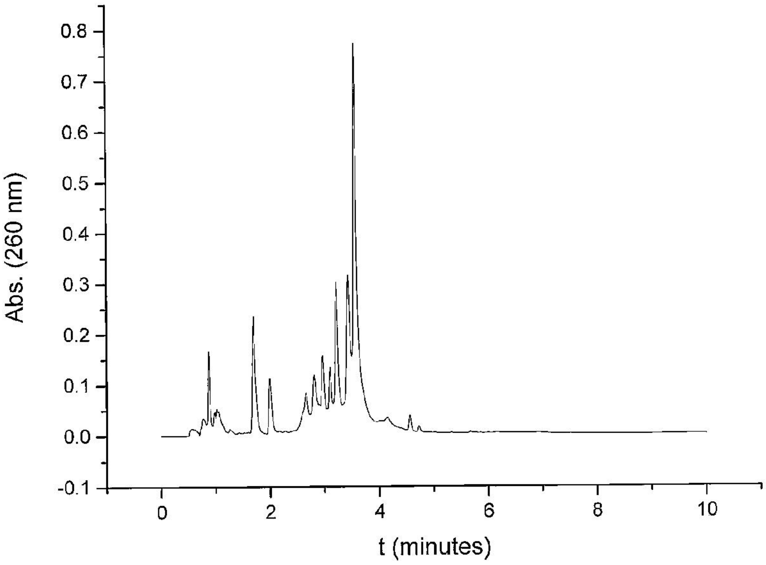

Figure S10. Mass spectrometry of compound **18**.

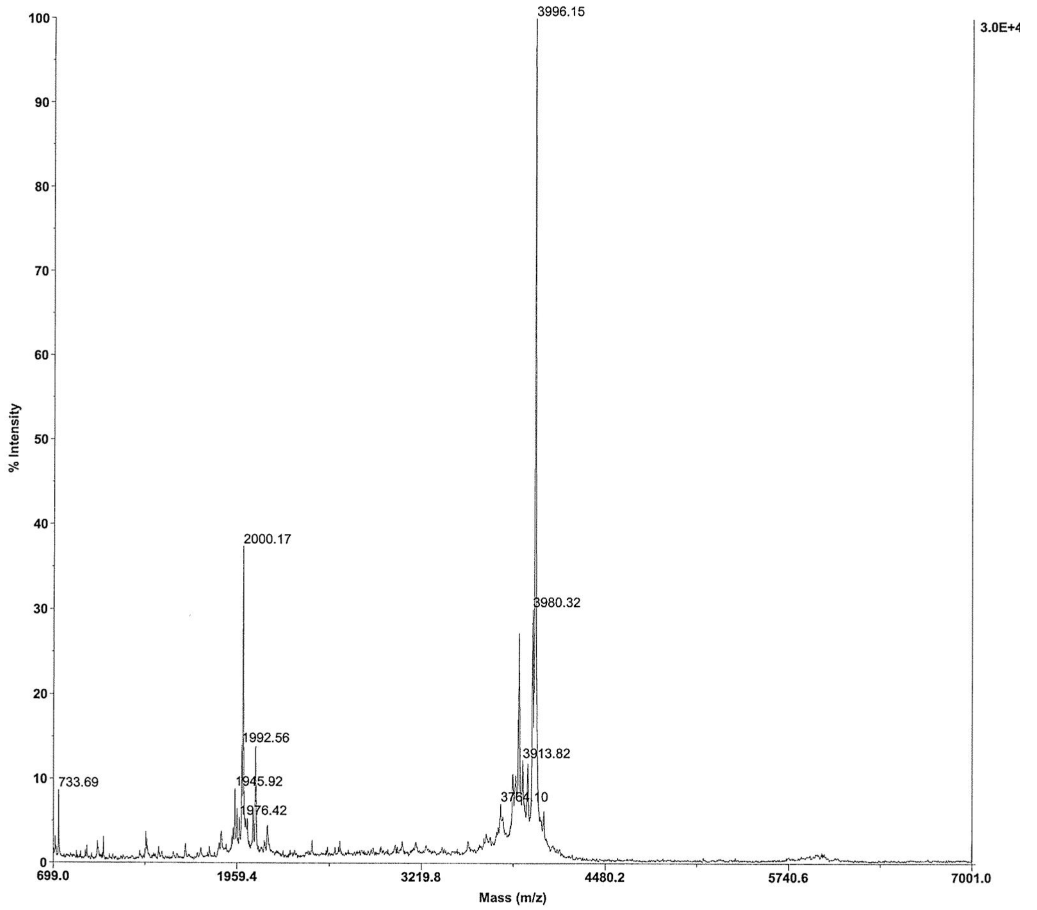

Mcalc.: 4372.08; Mfound: 4369.44

Figure S11. HPLC profile of *c-myc* peptide conjugate **19**.

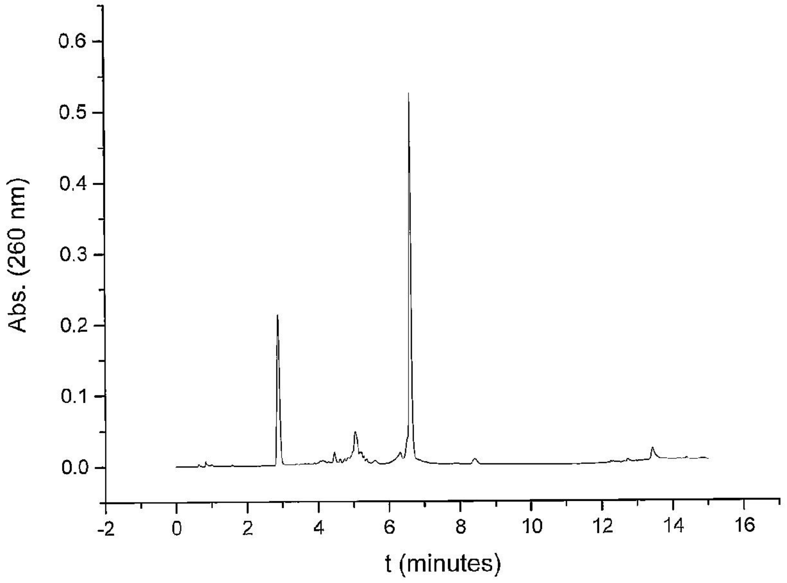

Figure S12. Mass spectrometry of *c-myc* peptide conjugate **19**.

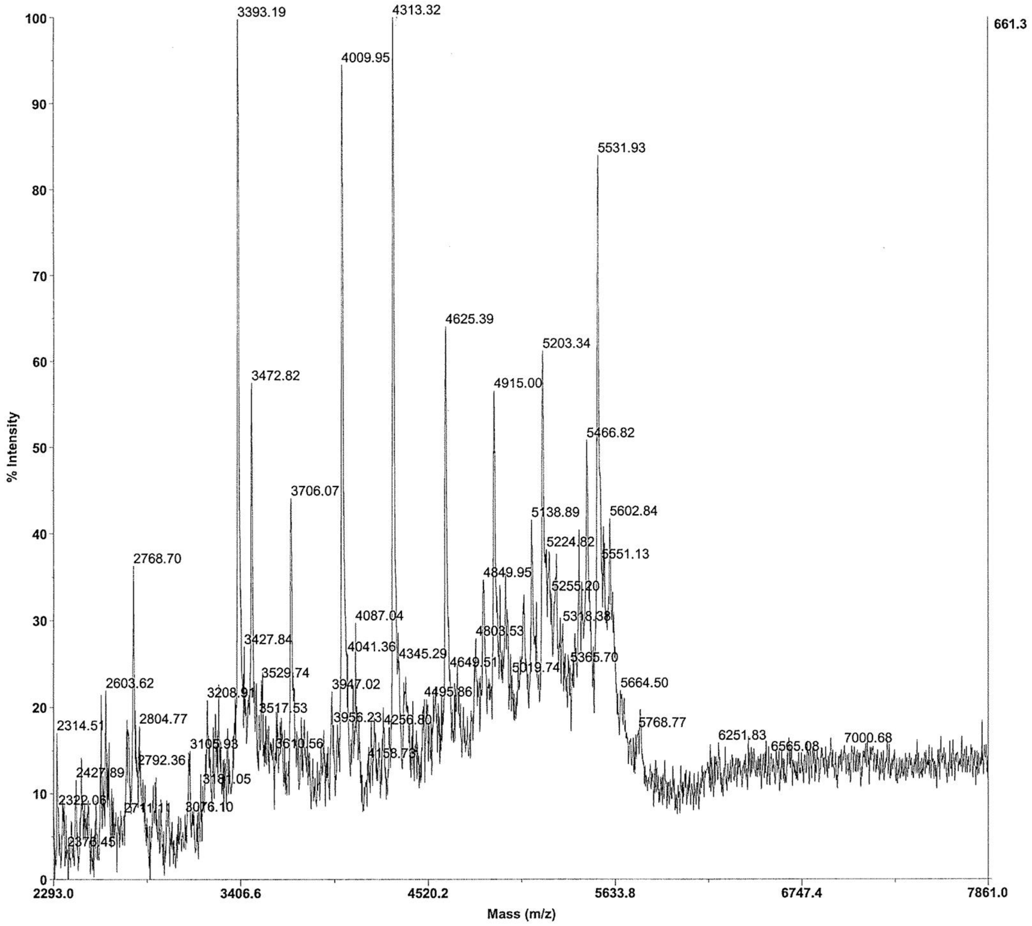

Mcalc.: 5529.90; Mfound: 5531.93

© 2011 by the authors; licensee MDPI, Basel, Switzerland. This article is an open access article distributed under the terms and conditions of the Creative Commons Attribution license (http://creativecommons.org/licenses/by/3.0/).
